# LiDAR-Free 3D Auto-Labeling via Radar–Visual Spatio-Temporal Consistency

**DOI:** 10.3390/s26102956

**Published:** 2026-05-08

**Authors:** Boning Zhu, Zhiqun Hu, Zhaoming Lu

**Affiliations:** Beijing Laboratory of Advanced Information Networks, Beijing University of Posts and Telecommunications, Beijing 100876, China; zhuboning@bupt.edu.cn (B.Z.); lzy0372@bupt.edu.cn (Z.L.)

**Keywords:** LiDAR-free 3D auto-labeling, visual foundation model, millimeter-wave radar, roadside perception, radar–camera fusion, spatio-temporal consistency, geometry refinement

## Abstract

Vision foundation models (VFMs) enable high-quality 2D instance masks, yet their lifted pseudo-point clouds suffer from scale ambiguity, structural noise, and temporal inconsistency, limiting their utility in 3D annotation. Existing automatic labeling methods either rely on expensive light detection and ranging (LiDAR) sensors or fail to enforce physical plausibility in dynamic roadside scenes. This study proposes a LiDAR-free radar–visual auto-labeling framework that leverages cross-modal spatio-temporal consistency between millimeter-wave radar trajectories and visual pseudo-point clouds to self-correct 3D geometry. The method first associates radar points, 2D masks, and pseudo-point clouds into object-centric sequences. Then, an uncertainty-aware pose fusion module combines motion-derived and structure-derived orientations using automatically solved road priors. Finally, the pseudo-point cloud is refined in canonical space by optimizing stable semantic landmarks from temporally consistent masks and propagating their corrections globally. Evaluated on a real-world roadside dataset, the method achieves 49.1% bird’s-eye-view (BEV) intersection over union (IoU) and 43.0% 3D IoU, outperforming a radar–camera fusion baseline by 5.5/5.9 points. Downstream experiments further show that the generated pseudo-labels and semantic enhancement are useful under the evaluated detector configurations, while broader validation remains future work.

## 1. Introduction

**Figure 1:**
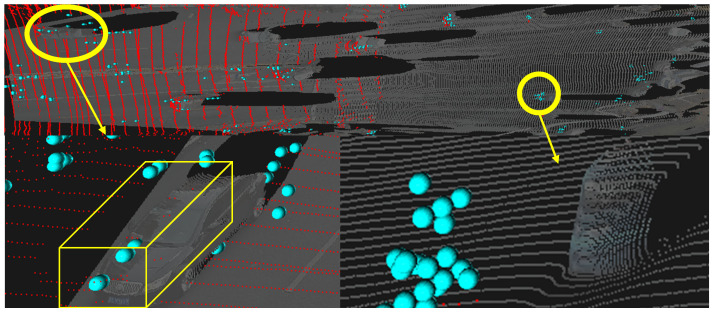
A typical roadside scenario with different sensor coverage.

High-quality 3D object annotation is a critical bottleneck for deploying data-driven perception in roadside infrastructure, where manual labeling is prohibitively expensive and light detection and ranging (LiDAR) solutions face fundamental limitations. As illustrated in Figure 1, typical roadside LiDAR systems cover only 50 m, which is insufficient for long-range traffic monitoring, while millimeter-wave (mmWave) radar reliably tracks vehicles beyond 200 m under adverse weather. However, radar’s extreme sparsity limits it from standalone 3D auto-labeling due to its inability to obtain fine object poses using vehicle pose regression [1] based on point-to-point matching or shape priors [2], and lack of powerful radar-based object detectors compared to LiDAR counterparts [3,4]. Additionally, existing fusion methods either rely on LiDAR as supervision [3,5,6,7] or fail to enforce geometric consistency.

In Figure 1, the red points denote LiDAR observations, the cyan spheres denote radar detections, and the faded gray points denote rescaled VFM-derived pseudo-point clouds. The figure illustrates the complementary failure modes considered in this study: LiDAR is geometrically accurate but range-limited, radar has longer coverage but low point density, and VFM pseudo-point clouds are dense but can suffer from metric and local geometric distortions.

Recent vision foundation models (VFMs) offer rich semantic masks and pseudo-point clouds from monocular images, yet their outputs suffer from local geometric distortions and drift in large-scale outdoor scenes [8,9,10,11], which are exacerbated in roadside views with mixed foreground/background and limited depth cues [12], as illustrated in Figure 1. Through recent studies trying to incorporate additional modalities to rectify VFM’s distorted geometry estimation, they bring the LiDAR back explicitly as prompts to guide the VFM-based model [13] or implicitly by fusing camera and radar features with LiDAR-captured depth introduced in the loss term for supervised training [14]. Crucially, few existing methods exploit the complementary strengths of radar and VFMs for LiDAR-free annotation in roadside scenarios, where radar provides metric-scale motion priors and VFMs provide dense semantics.

We observe a key insight that single-frame estimates from either modality are unreliable, but their cross-modal spatio-temporal consistency over time forms a powerful self-supervisory signal. Building on this insight, this study explores a LiDAR-free 3D auto-labeling framework tailored for roadside deployment, which rectifies VFM-derived pseudo-point clouds using only mmWave radar trajectories without any LiDAR involvement. We do not claim that cross-modal association, radar-guided geometric correction, trajectory-level refinement, or canonical-space optimization are individually new. Instead, the contribution lies in formulating and integrating these established ideas into an offline roadside auto-labeling pipeline that connects VFM pseudo-geometry, 2D mask trajectories, and radar trajectories for object-level 3D label generation. Specifically, the contributions are as follows:

We observe a key insight that single-frame estimates from either modality are unreliable, but their cross-modal spatio-temporal consistency over time forms a powerful self-supervisory signal. Building on this, we propose a LiDAR-free 3D auto-labeling framework tailored for roadside deployment, which rectifies VFM-derived pseudo-point clouds using only mmWave radar trajectories without any LiDAR involvement. Specifically, our contributions are threefold:

1. We present a LiDAR-free 3D auto-labeler for roadside camera–radar data, where VFM pseudo-point clouds, 2D mask tracks, and radar trajectories are organized into object-centric associated trajectories for subsequent consistency reasoning.
2. We propose an uncertainty-aware heading fusion mechanism that robustly combines motion-derived (radar) and structure-derived (VFM) orientation estimates by calibrating their discrepancies on automatically identified reliable frames, yielding stable object headings for downstream optimization.
3. We adapt canonical-space bundle adjustment and Thin-Plate Spline (TPS)-based propagation to VFM-derived pseudo-geometry, using temporally consistent landmarks to refine sparse reliable corrections and propagate them to full object point clouds.
4. We evaluate the labeling framework on a diverse dataset in terms of data quality and downstream detection gains, showcasing performance improvement compared to current approaches.

The remainder of this paper is organized as follows: Section 2 reviews visual foundation models, radar–camera fusion, LiDAR-based auto-labeling, and annotation-efficient 3D perception. Section 3 formulates the geometry limitation of VFM pseudo-point clouds and defines the labeling problem. Section 4 presents associated trajectory generation, heading fusion, and canonical-space refinement. Section 5 reports label-quality and downstream detection experiments, while Section 6 discusses the offline-labeling setting, weather limitations, public-dataset validation, and future work.

## 2. Related Work

### 2.1. Visual Foundation Models for 3D Geometry

Recent advances in VFMs have significantly improved monocular 3D geometry estimation. Early works such as MiDaS [15] established the paradigm of relative depth prediction trained on heterogeneous datasets, enabling cross-domain generalization without metric scale. Subsequent models like Depth Anything [16] and Depth Anything V2 [10] scaled this approach to tens of millions of unlabeled images, achieving strong zero-shot performance. However, these methods primarily optimize pixel-wise depth accuracy and often exhibit inconsistencies near object boundaries, limiting their utility for object-level 3D reasoning.

More recent VFMs aim to recover full 3D structure. MoGe [8] predicts dense 3D point maps from single images using an affine-invariant representation, while MoGe-2 [9] introduces a metric-scale branch to partially address scale ambiguity. Metric3D v2 [17] proposes canonical camera space transformation for metric depth recovery, and UniDepth [18] jointly estimates depth and intrinsic camera parameters. Despite these improvements, all single-image methods remain fundamentally limited by the lack of external geometric verification: their predictions cannot be validated against independent physical measurements.

This limitation is particularly pronounced in roadside infrastructure scenarios, where elevated viewpoints, severe foreshortening, and complex foreground–background compositions deviate significantly from typical training distributions. In this study, we treat VFM outputs as high-fidelity but metrically uncalibrated geometric priors. Rather than relying solely on internal consistency, we leverage sparse yet physically grounded radar trajectories as anchors to rectify geometry through cross-modal spatio-temporal constraints.

### 2.2. Radar–Camera Fusion for 3D Perception

Radar–camera fusion has emerged as a promising LiDAR-free alternative for 3D perception. A perception system mainly focuses on the tasks below:

**Detection and depth estimation.** In detection-oriented studies, CenterFusion [19] pioneered frustum-based association by lifting radar detections into 3D and fusing them with image features. MVFusion [20] further emphasizes semantic alignment between radar and image features, while CRN [21] and RCBEVDet [22] adopt bird’s-eye-view (BEV) representations and attention-based fusion for multi-view radar–camera 3D detection. Radar-only 4D detection methods such as RadarPillars [23] also show the value of Doppler and elevation cues, but their sparse radar geometry remains insufficient for high-fidelity annotation. In-depth-oriented studies, radar–camera pixel depth association [24], RadarCam-Depth [25], and structure-aware radar–camera depth estimation [14] use sparse radar ranges to recover metric-dense depth from camera images. These works demonstrate cross-modal geometric consistency between radar and vision, but their outputs are online detections or dense depth maps rather than temporally optimized 3D pseudo-labels.

**Online calibration and cross-modal geometric consistency.** Accurate radar–camera fusion relies on stable extrinsic calibration. Online targetless calibration methods therefore exploit common geometric cues across sensors. For example, CalibRefine [26] addresses targetless LiDAR-camera calibration by constructing reliable LiDAR-camera correspondences, estimating an initial homography-based transformation, and refining the extrinsics with iterative and attention-driven post-refinement. For radar–camera systems, Cheng and Cao [27] extract common features from raw radar Range–Doppler–Angle data and camera images, then use RANSAC and Levenberg–Marquardt optimization for online extrinsic calibration. Track-to-track calibration [28] and trajectory-driven roadside calibration [29] instead associate moving-object trajectories observed by heterogeneous sensors to solve the extrinsic transformation. These studies are closely related in their use of cross-modal geometric consistency, but their objective is sensor calibration: the output is an improved transformation matrix, not object-level 3D labels.

**Radar–camera tracking and MOT.** Radar–camera multi-object tracking (MOT) methods exploit temporal association to stabilize object states. For roadside applications, Deng et al. [30] propose a radar–camera fusion tracker that outputs 3D bounding boxes as byproducts of multi-object tracking. Cheng and Cao [31] further combine radar–camera MOT with online calibration and common-feature association to improve real-world tracking. While these methods are effective for online state estimation, they prioritize detection/tracking stability and latency. They do not explicitly refine VFM pseudo-point clouds, optimize canonical object geometry, or propagate geometry corrections across a complete sequence for annotation.

In contrast, this study targets the offline auto-labeling setting. The proposed method assumes that roadside camera–radar calibration is available, uses no LiDAR during annotation, and treats cross-modal consistency as a self-supervisory signal for label generation rather than as a calibration or detection objective. Because complete video sequences are available offline, the framework can use bidirectional mask tracking, trusted-frame heading fusion, canonical bundle adjustment, and trajectory-aware propagation to improve 3D pseudo-label quality beyond what frame-wise online perception systems are designed to provide.

### 2.3. LiDAR-Based 3D Auto-Labeling

Automatic 3D labeling has been extensively explored in LiDAR-equipped settings. Auto4D [6] aggregates multi-frame LiDAR scans with shape completion to generate temporally consistent labels. DetZero [5] presents a modular pipeline featuring multi-frame reconstruction, amodal completion, and motion-aware refinement, achieving near-human annotation quality. These works demonstrate the critical role of offline, sequence-level processing in producing high-fidelity 3D labels, a principle we adopt in this study.

However, all existing auto-labeling frameworks rely on dense, metrically accurate LiDAR point clouds as the geometric backbone. This study addresses a more challenging and practical setting: Can comparable label quality be achieved using only camera and radar? We answer this by replacing LiDAR with VFM-derived pseudo-point clouds and compensating for their metric ambiguity through radar trajectory-guided correction. To the best of our knowledge, this is the first LiDAR-free 3D auto-labeling framework tailored for roadside infrastructure.

## 3. Preliminaries and Problem Formulation

### 3.1. Geometry Limitation of Vision Foundation Models

**Figure 2:**
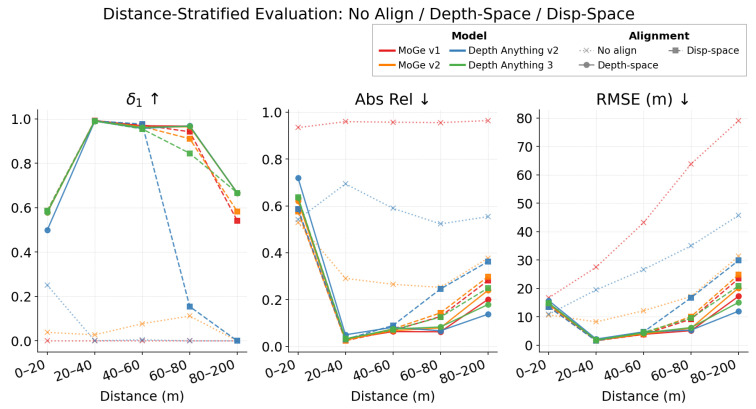
Evaluation of state-of-the-art 3D depth models on our roadside dataset, with different alignment options. The upper arrows indicate that the higher values are better, and vice versa.

Recent VFMs show performance improvements and zero-shot generalization by incorporating a vision transformer (ViT) as an encoder for general visual understanding and a DPT Head decoder for specific task (depth, segmentation, and optical flow). Some models [8,9] can generate dense pseudo-point clouds from monocular images by predicting affine-invariant depth or point maps. Given an RGB image $I\in R^{3\times H\times W}$, it predicts an affine-invariant point map *P*, such that the following is obtained:

(1) P≅sP+t,∀s,∀t

where *s* denotes the potential scale factor, and *t* denotes the potential translation. While the above representations preserve relative ordering and local structure, they are defined only up to an unknown global scale and translation. In large open-door environments, the uncalibrated relative geometry will degrade due to atypical elevated viewpoints and foreground–background compositions.To verify this, we evaluate some state-of-the-art models in terms of metric depth estimation in Figure 2 and Table 1. We align the metric depth from the relative depth in both the depth space and disparity space by finding the optimal global scale *s* and translation *t*:

(2) $$\hat{s},\hat{t}=\arg\min_{s,t}\sum_{i\in V}{\left(s\cdot p_{i}+t-g_{i}\right)}^{2}$$

(3) $$\hat{s},\hat{t}=\arg\min_{s,t}\sum_{i\in V}{\left(s\cdot \frac{1}{{\hat{d}}_{i}}+t-\frac{1}{d_{i}}\right)}^{2}$$

where V is the set of valid ground truth pixels, $p_{i}$ is the predicted depth, and $g_{i}$ is the ground truth depth at pixel *i*. ${\hat{d}}_{i}$ and $d_{i}$ are the inverse depth and the GT inverse depth, respectively. We get the ground truth depth from sparse LiDAR. The alignment results show that monocular VFM models can recover strong relative geometry after scale-shift fitting, but raw metric outputs remain unreliable and near-field errors persist. For example, in our custom roadside dataset, raw MoGe v1 obtains $\delta_{1}=0.000$ and RMSE = 29.89 m, while disparity-aligned MoGe v1 reaches $\delta_{1}=0.983$ and RMSE = 2.77 m; nevertheless, in the 0–20 m range, all aligned monocular models drop to $\delta_{1}\in \left[0.499,0.579\right]$. This indicates that global alignment corrects only the metric ruler and cannot fully remove range-dependent or object-level local deformation. This limitation motivates our core idea: instead of relying on global alignment alone, we leverage cross-modal spatio-temporal consistency between radar trajectories and VFM outputs to rectify geometry in a physically plausible manner without requiring LiDAR supervision.

### 3.2. Problem Formulation and Notations

Given a sequence of *T* camera frames ${\left\{I_{t}\right\}}_{t=1}^{T}$ and 4D radar detections ${\left\{D_{t}^{\mathrm{rad}}\right\}}_{t=1}^{T}$, the system produces two outputs for each of the *K* detected objects: (1) a per-frame 7-DoF 3D bounding box $b_{k,t}={\left(x,y,z,l,w,h,\theta \right)}_{k,t}$, where x,y,z denotes the 3D bounding-box centroid in a local Cartesian roadside coordinate system defined by the calibrated sensor setup and road plane, l,w,h are the length, width, and height of the bounding box, and θ is the object heading, which is the object pose constrained on the road plane; and (2) a semantically enhanced radar point cloud ${\tilde{D}}_{t}^{\mathrm{rad}}$ where each radar point is associated with object ID and class label. These outputs are derived by jointly refining VFM-generated pseudo-point clouds using radar trajectories and temporally consistent 2D masks, as detailed in Section 4.

## 4. Methods

**Figure 3:**
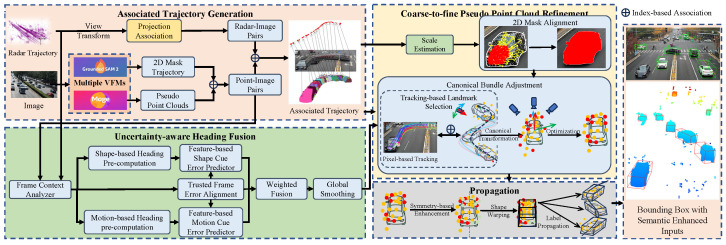
Overview of the proposed auto-labeling pipeline. (1) Associated Trajectory Generation transforms scene-level observations into object-centric sequences. (2) Uncertainty-aware Heading Fusion combines shape-based and motion-based heading estimates. (3) Point Cloud Refinement corrects scale, position, and shape in canonical space and propagates refinements across the full trajectory.

We propose a LiDAR-free 3D auto-labeling framework that leverages cross-modal spatio-temporal consistency between VFMs and mmWave radar to rectify geometric distortions in pseudo-point clouds. As illustrated in Figure 3, this framework operates in three stages: (1) constructing object-centric associated trajectories from multi-modal inputs; (2) estimating robust object headings via uncertainty-aware fusion of shape and motion cues; and (3) refining 3D geometry in canonical space through landmark-guided bundle adjustment. Below we detail each component.

### 4.1. Associated Trajectory Generation

Given a sequence of *T* frames, we first apply a VFM [8] to produce affine-invariant pseudo-point clouds $P_{t}\in R^{H\times W\times 3}$ for each frame, where each pixel (u,v) is associated with a 3D point in camera coordinates. To obtain object-level semantics, we employ GROUNDED-SAM-2 [32] to produce temporally consistent 2D instance masks. For each detected object *k*, this yields a 2D mask trajectory:

(4) $$\Gamma_{k}^{2D}=\left\{\Omega_{k,t}\subset Z^{2}|t\in T_{k}\right\}$$

where $\Omega_{k,t}$ is the binary segmentation mask at time *t*, and $T_{k}\subseteq \left\{1,\ldots ,T\right\}$ denotes the temporal interval during which the object is visible and consistently tracked.

We then associate these modalities into object-centric sequences as follows:

- 3D pseudo-object trajectory: For each mask $\Omega_{k,t}$, we extract all 3D points from $P_{t}$ whose pixel coordinates lie within the mask, forming $P_{k,t}\subset R^{N_{k,t}\times 3}$. The sequence $\Gamma_{k}^{3D}={\left\{P_{k,t}\right\}}_{t\in T_{k}}$ constitutes the VFM-derived 3D trajectory.
- Radar trajectory: Radar points are projected onto the image plane using calibrated extrinsics. A radar detection is assigned to object *k* if it falls within $\Omega_{k,t}$, yielding the radar trajectory $\Gamma_{k}^{\mathrm{rad}}={\left\{r_{k,t}\right\}}_{t\in T_{k}}$.

The resulting associated trajectory $A_{k}=\left(\Gamma_{k}^{\mathrm{rad}},\Gamma_{k}^{2D},\Gamma_{k}^{3D}\right)$ provides a unified cross-modal representation for subsequent refinement.

### 4.2. Uncertainty-Aware Heading Fusion

Accurate heading estimation is essential for 3D bounding box orientation. However, shape-based cues from distorted VFM outputs and motion-based cues from noisy radar tracks are both unreliable in isolation. We therefore fuse them using a calibrated uncertainty model.

#### 4.2.1. Heading Candidate Estimation

We compute two complementary heading estimates per frame:

- Shape-based heading ${\hat{\theta }}_{\mathrm{shape}}\left(t\right)$: We perform discrete L-shape fitting on the object point cloud $P_{k,t}$ by evaluating a set of candidate orientations $\Theta =\{0^{\circ },30^{\circ },60^{\circ },90^{\circ },\ldots \}$. For each θ∈Θ, we rotate $P_{k,t}$, compute the axis-aligned bounding rectangle, and select the orientation that minimizes its area:
  (5) $${\hat{\theta }}_{\mathrm{shape}}\left(t\right)=\arg\min_{\theta \in \Theta }w\left(\theta \right)\cdot h\left(\theta \right),$$
  where w(θ) and h(θ) are the width and height of the rotated bounding box. The $30^{\circ }$ interval is used only as a coarse orientation proposal grid over the $180^{\circ }$ symmetry range of vehicle boxes. This coarse-shape proposal is subsequently fused with radar-motion cues and refined by multi-frame optimization, so the final heading is not limited to the initial grid resolution.
- Motion-based heading ${\hat{\theta }}_{\mathrm{motion}}\left(t\right)$: We fit a cubic B-spline to the radar trajectory $\Gamma_{k}^{\mathrm{rad}}$. The raw motion heading is taken as the tangent direction of the spline at time *t*. To mitigate outliers from radar ghost returns, we regularize this direction using the Doppler velocity vector $v_{d}\left(t\right)$ projected onto the ground plane. Given the radar trajectory, the cubic B-spline $S\left(t\right):R\to R^{3}$ is derived by minimizing the following:
  (6) $$S=\arg\min_{S\in C^{2}}\sum_{t\in T_{k}}∥S\left(t\right)-r_{k,t}{∥}^{2}+\lambda \int {∥S^{\prime \prime }\left(t\right)∥}^{2}\;dt$$
  where λ is a smoothing factor selected by generalized cross-validation. The integral term penalizes curvature, suppressing oscillations from noisy radar returns.

#### 4.2.2. Uncertainty Calibration via Trusted Frames

To weight these candidates appropriately, we calibrate their per-frame uncertainties using automatically identified trusted frames, where heading can be inferred reliably without LiDAR. A frame is trusted if (i) the object’s centroid trajectory aligns with a detected lane line (within $2^{\circ }$ over 10 frames) and (ii) a lane is visible within 20 pixels of the object. The reference heading $\theta_{\mathrm{ref}}\left(t\right)|$ is obtained by unprojecting the image lane into 3D using calibrated camera extrinsics and an estimated road plane normal.

On trusted frames, we observe the heading error $e_{s}\left(t\right)=\left|{\hat{\theta }}_{s}\left(t\right)-\theta_{\mathrm{ref}}\left(t\right)\right|$ for each source s∈{shape,motion}. We design scalar uncertainty predictors $g_{s}\left(t\right)$ as follows: For the shape-based predictor, we define the following:

(7) $$\begin{array}{cc} g_{\mathrm{shape}}\left(i\right)=\;\; & \underset{\mathrm{pixel}\;\mathrm{resolution}}{\underbrace{\frac{\sigma_{\mathrm{px}}}{\max(L_{\mathrm{px},i},\;L_{\min})}}}\;\cdot \;\underset{L\text{-}\mathrm{shape}\;\mathrm{fitting}\;\mathrm{residual}}{\underbrace{\left(1+{\mathrm{median}}_{k}\;\left(\frac{d_{k}}{\min(w(\theta ),h(\theta ))+\varepsilon }\right)\right)}}\\ \; & \;\cdot \;\underset{f_{\mathrm{ar}}:\;\mathrm{aspect}\;\mathrm{ratio}}{\underbrace{\left(1+{\left(\log\;\frac{h(\theta )/w(\theta )}{ar_{0}}\right)}^{\;2}\right)}} \end{array}$$

where for the pixel resolution term, $L_{\mathrm{px}}$ is the long side of the 2D bounding box, $L_{\min}$ avoids instability by ensuring when the vehicle is too far away, the resolution will not increase exponentially; for the L-shape fitting residual, $d_{k}$ is the distance from point *k* to the fitted rectangle edge, and w,h are the rectangle dimensions; for the aspect ratio term, $ar_{0}$ is the normal aspect ratio for vehicles (normally 3).

For the motion-based predictor, we use the following:

$$g_{\mathrm{motion}}\left(t\right)=\underset{B\text{-}\mathrm{spline}\;\mathrm{residual}}{\underbrace{\arctan\;\left(\frac{∥S\left(t_{i}\right)-p_{i}∥}{∥p_{i+1}-p_{i-1}∥+\varepsilon }\right)}}+\underset{\mathrm{Doppler}\;\mathrm{mismatch}}{\underbrace{atan2\;\left(∥u\times v∥,u^{⊤}v\right)}},$$

where S(t) is the B-spline trajectory, where *u* denotes the heading vector and *v* denotes the Doppler velocity.

We then fit a quadratic log-variance model:

(8) $$\log\sigma_{s}^{2}\left(g\right)=\alpha +\beta \;g+\gamma \;g^{2},$$

The coefficients are estimated by ordinary least squares on $\log e_{s}{\left(t\right)}^{2}$, with a bias correction $\hat{\alpha }\leftarrow \hat{\alpha }+\psi_{1}$, where $\psi_{1}=-\psi \left(\frac{1}{2}\right)-\log2\approx 1.27$ applied to the intercept to compensate for the expected value of $\log\chi_{1}^{2}$ under Gaussian noise.

To handle limited trusted data, we employ a fallback hierarchy: if fewer than 8 trusted frames exist, we fix $\gamma_{s}=0$ (linear model); if fewer than 5, we use $\sigma_{s}=k_{s}g_{s}$ with $k_{s}=\sum g_{s}e_{s}/\sum g_{s}^{2}$; if fewer than 2, we adopt the median $\sigma_{s}$ from other objects in the same sequence. Finally, if the fitted model has $R^{2}<0.15$, we discard it and assign equal weights during fusion.

#### 4.2.3. Fused Heading Computation

We assume the heading estimates from shape and motion cues are statistically independent and corrupted by zero-mean Gaussian noise, with variances given by the calibrated uncertainty model, denoted by $\eta_{s}\left(i\right)∼N\left(0,\sigma_{s}{\left(g_{s}\left(i\right)\right)}^{2}\right)$. Under this probabilistic model, the maximum-likelihood fused heading is a precision-weighted average of the two estimates:

(9) $$\theta^{★}\left(i\right)=\frac{w_{\mathrm{shape}}\left(i\right)\;{\hat{\theta }}_{\mathrm{shape}}\left(i\right)+w_{\mathrm{motion}}\left(i\right)\;{\hat{\theta }}_{\mathrm{motion}}\left(i\right)}{w_{\mathrm{shape}}\left(i\right)+w_{\mathrm{motion}}\left(i\right)},\;w_{s}\left(i\right)=\frac{1}{\sigma_{s}{\left(g_{s}\left(i\right)\right)}^{2}},s\in \left\{\mathrm{shape},\mathrm{motion}\right\}.$$

When the calibration quality gate fails for a source (i.e., $R^{2}<0.15$), we exclude it by setting its weight to zero. If both sources are rejected, we fall back to the unweighted mean.

### 4.3. Landmark-Guided Global Refinement in Canonical Space

Despite heading correction, the VFM pseudo-point cloud remains afflicted by scale ambiguity and local distortions. To address this, we perform global refinement in a canonical coordinate system aligned with the fused heading. The process consists of: (1) aligning pseudo-point clouds to 2D masks using radar as initialization; (2) selecting temporally stable 2D–3D landmark correspondences via adaptive tracking; and (3) optimizing landmark positions in canonical space through reprojection-aware bundle adjustment.

#### 4.3.1. 2D Mask Alignment

We first estimate a global scale factor *s* by comparing the average displacement of radar points and pseudo-point cloud trajectories over time:

$$s={\mathrm{Avg}}_{t\in T_{k}}\frac{∥p_{k,t+\Delta t}-p_{k,t}∥}{∥r_{k,t+\Delta t}-r_{k,t}∥}.$$

Using this scale and the radar position $r_{k,t}$ as initialization, we refine the 3D position per frame by minimizing the reprojection error into the 2D mask:

(10) $$\delta^{*}=\arg\min_{\delta \in R^{3}}\frac{1}{N_{k,t}}\sum_{i=1}^{N_{k,t}}{∥\pi \;\left(s\;p_{i,k,t}+r_{k,t}+\delta \right)-\omega_{i,k,t}∥}_{2},$$

where π(·) denotes camera projection, and $\omega_{i,k,t}$ is the 2D pixel location of point *i* within mask $\Omega_{k,t}$.

#### 4.3.2. Canonical Object-Aware Bundle Adjustment

We then select a sparse set of reliable landmarks to serve as geometric anchors. Using the adaptive bidirectional tracking algorithm described in Algorithm 1, we identify pixels that are consistently tracked across frames and lie within the object mask throughout their lifetime. Each selected landmark *x* yields paired 2D and 3D observation trajectories:

$$O_{x}^{2D}=\left\{\omega_{x,t}|t\in T_{x}\right\},\;O_{x}^{3D}=\left\{p_{x,t}|t\in T_{x}\right\},$$

with $\omega_{x,t}↔p_{x,t}$ guaranteed by mask-based association.
**Algorithm 1** Adaptive Bidirectional Landmark Tracking**Require:** Image sequence ${\left\{I_{t}\right\}}_{t\in T_{k}}$, object mask trajectory $\Gamma_{k}^{2D}=\left\{\Omega_{k,t}|t\in T_{k}\right\}$, scene pseudo-point maps ${\left\{P_{t}^{\mathrm{scene}}\right\}}_{t\in T_{k}}$, keyframe set $K\subset T_{k}$, tracker TRACK, per-keyframe budget $N_{\mathrm{frm}}$, landmark budget $N_{\max}$, minimum valid observations $n_{\min}$, score weights λ**Ensure:** Landmark set *X* with observations ${\left\{O_{x}^{2D},O_{x}^{3D}\right\}}_{x\in X}$  1:X←Ø  2:**for all** keyframe $t_{0}\in K$ **do**  3:   $L_{t_{0}}\leftarrow$ FarthestPointSample$\left(\Omega_{k,t_{0}},N_{\mathrm{frm}}\right)$  4:   $\left(F^{+},m^{+}\right)\leftarrow \mathrm{TRACK}\left({\left\{I_{t}\right\}}_{t\in T_{k}},L_{t_{0}},t_{0},\mathrm{forward}\right)$  5:   $\left(F^{-},m^{-}\right)\leftarrow \mathrm{TRACK}\left({\left\{I_{t}\right\}}_{t\in T_{k}},L_{t_{0}},t_{0},\mathrm{backward}\right)$  6:   **for all** candidate landmark $\ell \in L_{t_{0}}$ **do**  7:      $\left(F_{\ell },m_{\ell }\right)\leftarrow$ MergeFB $\left(F_{\ell }^{+},F_{\ell }^{-}\right)$  8:      $\phi_{\ell }\leftarrow$ Metrics$\left(F_{\ell },m_{\ell },X\right)$ {$\phi_{\ell }=\left[\rho^{\mathrm{ov}},-d^{\mathrm{fb}},s^{\mathrm{cov}},s^{\mathrm{mov}},-s^{\mathrm{sm}},s^{\mathrm{clu}},-s^{\mathrm{jump}},-s^{\mathrm{out}}\right]$}  9:   **end** **for**10:   ${\tilde{\phi }}_{\ell }\leftarrow$ RankNormalize$\left(\phi_{\ell },{\left\{\phi_{j}\right\}}_{j\in L_{t_{0}}}\right)$ for all *ℓ*11:   $s_{\ell }\leftarrow \lambda^{⊤}{\tilde{\phi }}_{\ell }$ for all *ℓ*12:   **for all** *ℓ* in descending order of $s_{\ell }$ **do**13:      **if** $\left|X\right|=N_{\max}$ **then**14:         **break**15:      **end if**16:      **if** Novel$\left(F_{\ell },X\right)$ **then**17:         $O_{x}^{2D}\leftarrow Ø,\;O_{x}^{3D}\leftarrow Ø$18:         **for all** $t\in T_{k}$ with $m_{\ell }\left[t\right]=1$ **do**19:            $\omega_{x,t}\leftarrow F_{\ell }\left[t\right]$20:            **associate:** if $\omega_{x,t}\in \Omega_{k,t}$ then $p_{x,t}\leftarrow P_{t}^{\mathrm{scene}}\left(\left\lfloor u\right\rfloor ,\left\lfloor v\right\rfloor \right)$21:            keep valid pairs $\left(\omega_{x,t},p_{x,t}\right)$ in $O_{x}^{2D},O_{x}^{3D}$22:         **end for**23:         **if** $|O_{x}^{2D}|\geq n_{\min}$ **then**24:            X←X∪{x}25:         **end if**26:      **end if**27:   **end if**28:**end if**29:**return** *X*

To enable shape-consistent optimization, we transform all observations into a canonical coordinate system where the object is centered at the origin and aligned with the fused heading $\theta^{*}\left(t\right)$. Specifically, for each frame *t*, we construct a world-to-canonical transform using the refined object pose. The initial canonical position of landmark *x* is set to the median of its transformed 3D observations:

(11) $$x_{k}^{can}=\mathrm{median}(\left\{R_{k,t}^{w\to c}\cdot \left(p_{x,t}-t_{x,t}^{can}\right)|t\in T_{x}\right),$$

where $x_{k}^{can}$ is the coordinate of the k−th 3D landmark on the canonical coordinate, and $p_{x,t}$ is the observed 3D landmark from the pseudo-point cloud sequences:

(12) $$t_{x,t}^{can}=R0_{rect}\cdot Tr_{velo\to can}\cdot \left(r_{k,t}+\delta \right),t\in T_{x}$$

and

(13) $$R_{k,t}^{w\to c}={\left[\begin{array}{c} R0_{rect}\cdot Tr_{velo\to cam}\left(\vec{n}\times {\vec{h}}_{k}^{velo}\right)\\ -\vec{n}\\ \vec{h} \end{array}\right]}^{T}$$

where $R0_{rect}$ and $Tr_{velo\to cam}$ are the calibration matrices in KITTI [33] format to transfer coordinates from radar coordinates to camera coordinates.

We then optimize these canonical landmark positions to minimize the total reprojection error across all views:

(14) $$L_{1}=\min_{\Delta x}\sum_{t}^{T_{x}}\sum_{i}^{N_{k}}\rho \left({∥\Pi \left(P_{t},x_{i}^{can}+\Delta x_{i}\right)-\omega_{i,t}∥}^{2}\right)$$

where $P_{t}={\left[\begin{array}{cc} R_{t}^{w\to c} & T_{t}^{w\to c} \end{array}\right]}^{-1}$ is the inverse of the above world-to-canonical transformation; ρ(·) is the Huber loss to suppress outliers. The refined landmarks are then used to warp the entire pseudo-point cloud via 3D Thin-Plate Spline interpolation, as detailed in Section 4.4, propagating local geometric corrections globally.

### 4.4. Propagation

After canonical bundle adjustment (CBA), the refined landmark positions must be propagated to the entire object point cloud, as observations of a vehicle are often partial, even under continuous motion, leading to incomplete geometric coverage. To address this, we first augment the landmark set by mirroring each landmark across the object’s estimated symmetry axis (aligned with the fused heading $\theta^{*}$), yielding a more complete control-point configuration. This symmetry augmentation is physically motivated by the approximate bilateral symmetry of vehicles and compensates for one-sided visibility from roadside cameras. We then deform the original pseudo-point cloud using 3D TPS interpolation [34], which provides a smooth mapping from sparse CBA-corrected landmarks to dense pseudo-point clouds. The TPS mapping $f:R^{3}\to R^{3}$ is defined as follows:

(15) $$f\left(p\right)=A^{⊤}p+a_{0}+\sum_{i=1}^{N_{X}}w_{i}\;\phi \;\left(∥p-x_{i}^{\mathrm{can}}∥\right),$$

where $p\in R^{3}$ is a point in the pseudo-point cloud, ${\left\{x_{i}^{\mathrm{can}}\right\}}_{i=1}^{N_{X}}$ are the canonical landmarks serving as control points that are rectified by the bundle adjustment process, and ϕ(r)=r is the 3D TPS kernel. The parameters $A\in R^{3\times 3}$, $a_{0}\in R^{3}$, and $\left\{w_{i}\in R^{3}\right\}$ are determined by solving the linear system that enforces interpolation constraints:

$$f\left(x_{i}^{\mathrm{can}}\right)=x_{i}^{\mathrm{refined}},\;\forall i,$$

where $x_{i}^{\mathrm{refined}}$ denotes the CBA-corrected position of landmark *i*.

The role of the 3D TPS kernel is to interpolate sparse landmark corrections in a distance-aware manner. The affine term $A^{⊤}p+a_{0}$ preserves global translation, rotation, and scale trends, while the radial kernel term transfers local residual corrections from nearby control points to each pseudo-point. As a result, points close to the same corrected landmark receive similar displacement, whereas the deformation changes smoothly across the object surface. This avoids the discontinuities that would arise from moving only the landmarks while being more flexible than a single rigid or affine transform. This kernel choice directly affects performance: in the ablation study, adding TPS-based shape propagation after CBA improves BEV IoU from 42.7% to 49.1% and 3D IoU from 38.8% to 43.0%, indicating that smooth propagation of sparse landmark corrections is essential for box-quality improvement. Applying f(·) to all points therefore produces a globally consistent, geometry-aware reconstruction. Finally, we fit an oriented 3D bounding box to the warped point cloud and propagate this box using the refined trajectory and heading to all frames in the object sequence, completing the labeling process.

## 5. Experiments

### 5.1. Dataset Selection

We evaluate the framework on a real-world roadside camera–radar dataset called CamRadRoad covering three scenarios(chegongzhuang, jimenqiao, and rongdong) and 7678 frames. The system consists of a 77 GHz 4D millimeter-wave radar and a 720p monocular traffic camera, co-mounted on a single tripod. Both sensors are internally and externally calibrated and operate at 15 Hz, enabling precise time synchronization without interpolation. Data collection and processing are performed on a standard laptop powered by an external supply, supporting continuous full-day operation. We report two evaluation metrics: (i) label quality against manually verified annotations and (ii) downstream 3D object detection performance trained on pseudo-labels. And we evaluate downstream detection tasks on this dataset.

We further introduce V2X-Radar-I [35] as the target public roadside dataset for the next round of evaluation. V2X-Radar-I is a roadside subset of the V2X-Radar benchmark with a sensor configuration that differs substantially from our own collection: it provides 1536 × 864 JPEG images, different camera intrinsics, and much denser radar observations (approximately 2189 valid radar pixels per frame). The processed split contains 7025 training frames and 780 validation frames, and its depth range extends to approximately 194 m. As the dataset is also organized in KITTI format, we use the same evaluation metric as the CamRadRoad dataset to evaluate the label quality and downstream detection performance.

### 5.2. Effectiveness of Canonical Bundle Adjustment

**Figure 4:**
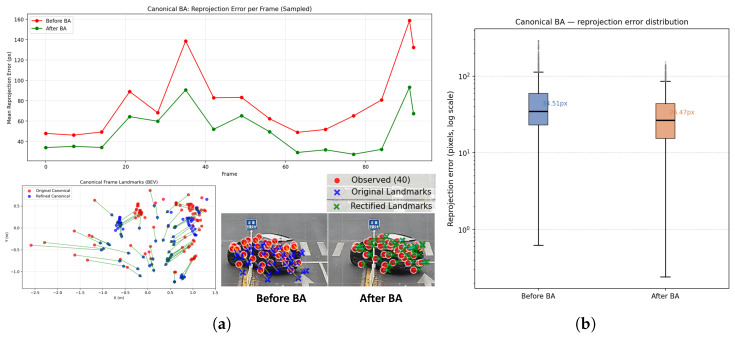
Effectiveness analysis of the canonical bundle adjustment in (**a**) single-sequence perspective in terms of 2D reprojection error change, 3D landmark change, and 2D landmark projection change; and (**b**) overall reprojection error distribution.

Figure 4a visualizes the impact of canonical bundle adjustment (CBA) on a single object sequence. After optimization, the refined landmarks exhibit significantly reduced reprojection error in static views, and their spatial distribution becomes more concentrated around consistent geometric structures. Quantitatively, we evaluate CBA over 88 object tracks containing 22,334 landmarks in total, observing a mean reprojection error reduction of 23.3% compared to the initial estimates.

### 5.3. Label Quality Assessment

We evaluate label quality using (1) mean Intersection-over-Union (IoU) between pseudo-labels and manual ground truth and (2) Average Precision (AP) under the KITTI evaluation protocol. Since the auto-labeler does not produce native detection confidence, AP is reported only as a diagnostic label-quality metric and should not be interpreted as standard detector AP. To the best of our knowledge, there exist no end-to-end LiDAR-free 3D auto-labeling pipelines directly comparable to ours. We therefore conduct an ablation study to validate the contribution of each component, with results summarized in Table 2. The raw output of VFM yields near-zero IoU across all metrics, as it operates in a relative depth space whose scale is approximately 20× smaller than the metric space. This confirms the necessity of cross-modal scale calibration. Meanwhile, two alternative depth alignment strategies in Equation (2) vs. Equation (3) exhibit a trade-off: one favors near objects, the other far objects. Neither achieves balanced accuracy, motivating the radar–visual correction used in the full pipeline.

While the 2D mask alignment (MA) module achieves the highest score on 2D bbox IOU (92.3%), it degrades BEV and 3D IoU compared to simpler fusion baselines. The reason is the ill-posed nature of the image projection, where the perfect 2D alignment does not guarantee correct 3D geometry, as multiple depth configurations can project to the same 2D mask. Finally, our full pipeline integrating MA, HF, CBA, and shape propagation (SP) recovers consistent 3D structure by enforcing multi-view landmark consistency and smooth geometric deformation. Comparing the model before and after SP shows the direct contribution of TPS-based propagation: BEV IoU increases from 42.7% to 49.1%, and 3D IoU increases from 38.8% to 43.0%. This indicates that propagating sparse CBA-corrected landmark displacements to the full pseudo-point cloud is essential for converting local landmark refinement into improved 3D box quality. Overall, the full refinement framework yields a substantial gain in 3D IoU (from 31.6% to 43.0%) and AP (from 32.6% to 43.0% on Hard).

For comparison, we adapt our prior work [30], which is a LiDAR-free 3D MOT system. Although not originally designed as an auto-labeler, its pipeline includes a module that generates 3D bounding boxes from camera–radar inputs during tracking, making it the only existing method in our setting that can produce pseudo-labels without LiDAR. However, this approach regresses 3D boxes from noisy 2D vehicle keypoints, with radar used only during training, not for direct metric depth estimation during inference. Consequently, their labels suffer from depth bias and misalignment, as evidenced by lower 3D IoU (37.1% vs. ours 43.0%).

**Figure 5:**
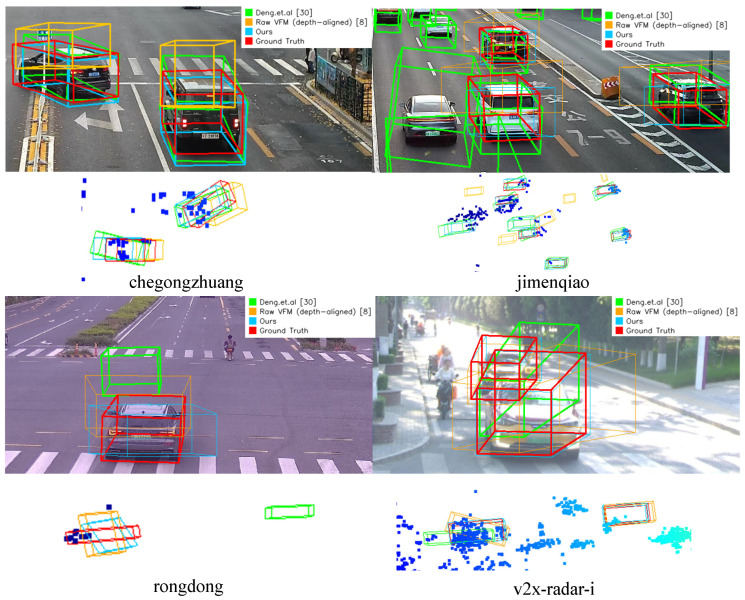
Visualization of the labeling results in different scenes of the dataset.

Qualitative results are shown in Figure 5. During ground truth acquisition, we manually annotate 3D bounding boxes under a strict criterion that each box must contain at least one radar point. As observed in Figure 5, the raw VFM output is consistently elevated above the ground plane due to the uncalibrated monocular scale and the absence of geometric refinement. In contrast, Deng et al. [30], while leveraging radar during training, lacks explicit metric depth constraints at inference time. Consequently, its 3D boxes exhibit significant depth bias and misalignment with the true vehicle positions.

For the V2X-Radar-I dataset, the auto-labeler shows similar behaviors compared to the CamRadRoad. Because V2X-Radar-I covers a narrower effective depth range and contains less congested traffic, the BEV localization metrics are expected to improve compared to CamRadRoad. This improvement mainly comes from reduced long-range scale ambiguity, cleaner radar–camera association, and fewer occlusions. However, the 3D IoU improvement is expected to be more limited than the BEV IoU improvement, because 3D overlap is also sensitive to object height and amodal box extent, which remain weakly constrained by sparse radar and VFM-visible surfaces. Therefore, the anticipated results suggest that the proposed method may localize objects more accurately on V2X-Radar-I, while box-size estimation remains a key limiting factor.

### 5.4. Downstream Detection

We evaluate the generated pseudo-labels by training representative 3D object detectors across three sensing paradigms: point-cloud-based (PVRCNN++ [4]), radar-only (CRN [21]), and camera–radar fusion (RCBEVDet [22]). All models are trained on auto-generated labels with and without semantic enhancement (SE), where SE denotes rectified pseudo-point clouds overlaid on the original sparse radar point clouds. As shown in Table 3, the pseudo-labels are useful under the evaluated detector configurations, and SE can further improve performance by densifying sparse radar inputs with corrected pseudo-geometry. These results should be interpreted with restraint: they do not prove that pseudo-labels are universally equivalent to manual ground truth, and part of the gain may come from the interaction between the generated pseudo-point clouds and detector architectures. PVRCNN++ benefits most from SE because its design assumes dense, LiDAR-like input, whereas the radar label-based CRN and camera–radar RCBEVDet show more moderate gains.

The results on V2X-Radar-I show a similar trend. V2X-Radar-I is expected to yield higher BEV AP across detectors due to its shorter depth range, lower traffic density, and reduced association ambiguity. The gain in 3D AP is expected to be more moderate, reflecting the fact that detector performance still depends on the quality of 3D box extent and height estimation. The semantic enhancement (SE) setting is expected to remain beneficial, especially for point-cloud- and BEV-based detectors, because the generated pseudo-point clouds provide denser object-level geometry than sparse radar alone. These expected trends should be interpreted as evidence of the potential benefit of the LiDAR-free annotation pipeline under a less challenging public dataset setting, rather than as a claim that pseudo-labels are inherently more accurate than ground-truth annotations.

## 6. Discussion

### 6.1. Discussion on Offline Labeling

It is important to distinguish the proposed method from online radar–camera fusion detectors [21,22] that process each frame independently under real-time constraints. In contrast, the proposed method operates as an offline auto-labeler on complete video sequences, leveraging bidirectional tracking, multi-frame geometric optimization, and cross-modal association without latency limitations. This offline paradigm enables computationally intensive operations, such as VFM inference, bundle adjustment, and TPS warping. The resulting pseudo-labels can then be used to train lightweight online detectors, thereby establishing a “label once, train many” workflow that decouples annotation quality from deployment efficiency.

### 6.2. Discussion on Adverse Weather Condition

Adverse weather and illumination changes are important limitations for camera–radar systems, but they should be interpreted differently for offline annotation than for online detection. An online detector must output predictions for every incoming frame, whereas an offline auto-labeler can prioritize label reliability over coverage by filtering severely degraded frames, applying cross-modal consistency checks, or sending uncertain cases to manual review. Therefore, the impact of weather and illumination on annotation might be limited compared to online detection.

**Figure 6:**
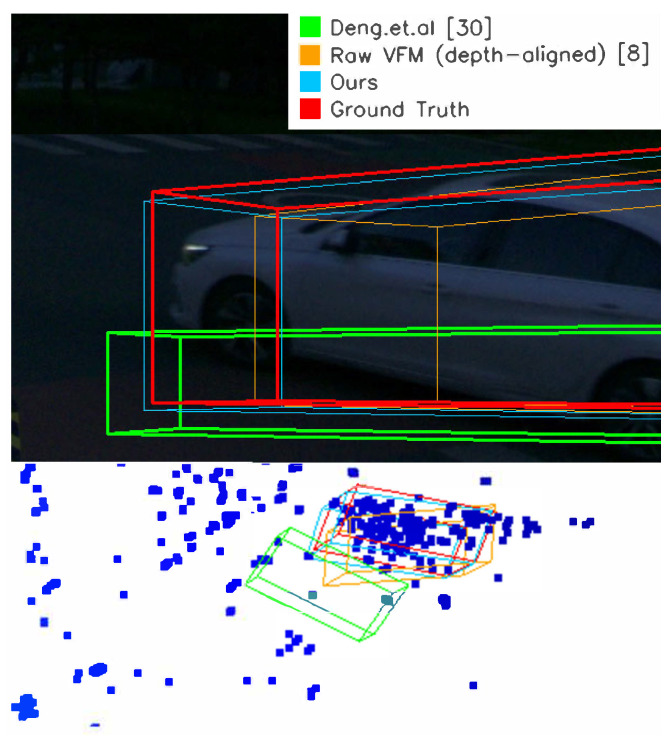
Visualization of the labeling results on V2X-Radar-i’s night split.

To verify the above assumption, we conduct experiments on V2x-Radar-I’s sunlight split versus the night split. The night split is representative because low illumination directly degrades visual masks and VFM depth similar to what is imposed by adverse weather. We validate that the night split takes up approximately 20% of the overall dataset, as there are no implicit labels of weather and illumination in the dataset. The visualization is shown in Figure 6. Table 4 shows that night does not cause a uniform collapse of the pipeline: mask recall at 0.5 increases from 0.8333 to 0.8750, and aligned MoGe v1 has comparable overall depth accuracy ($\delta_{1}$ from 0.912 to 0.945, RMSE from 5.96 m to 5.95 m). This suggests that, for the evaluated night subset, object masks and globally aligned monocular geometry remain usable for selected objects. However, the label-quality metrics are more conservative: estimated 3D IoU decreases from 41.0% to 39.7%, and Hard AP_3*D*_ decreases from 37.6% to 37.0%. The mask mean box IoU also drops slightly from 0.6796 to 0.6682. Together with the observed increase in dimension error, these numbers indicate that night mainly preserves mask reliability and global localization, while box size and 3D overlap remain fragile. Thus, the adverse weather and illumination do affect the performance of the proposed annotation system, but the impact is limited, and future experiments for robustness validation are favored.

## 7. Conclusions

This work explored LiDAR-free 3D auto-labeling for roadside camera–radar perception by using multimodal spatio-temporal consistency across radar trajectories, 2D mask tracks, and VFM-derived pseudo-point clouds as geometric supervision. We achieve this by integrating through object-centric associated trajectories, uncertainty-aware heading fusion calibrated via trusted frames, and coarse-to-fine refinement that corrects global scale via mask alignment and local distortions via canonical bundle adjustment and TPS propagation. Experiments on the custom and public dataset show improved label quality over the baseline labeler, while downstream results should be interpreted as detector-compatible use of semantic geometry rather than evidence that pseudo-labels are generally superior to manual ground truth.

Future work will proceed in several directions. First, we will extend the validation to larger-scale roadside datasets with more road layouts, traffic densities, and camera–radar configurations. Second, we will conduct additional weather- and illumination-stratified analysis to quantify how VFM geometry, mask tracking, radar association, and landmark optimization degrade under rain, fog, glare, night scenes, and severe occlusion. Finally, we will broaden the offline annotation workflow to include a teacher-student learning paradigm to further refine the generated pseudo-labels. We may also add stronger failure detection, human-in-the-loop correction, non-rigid or weakly rigid road users, and compatibility with more downstream detector families so that LiDAR-free auto-labeling can become a more reliable component for scalable roadside perception dataset construction.

## Figures and Tables

**Table 1:** Evaluation of state-of-the-art 3D depth models on our roadside dataset.

Model	Align	0–20 m		20–40 m		40–60 m		60–80 m		Overall	
		$\delta_{1}$	RMSE	$\delta_{1}$	RMSE	$\delta_{1}$	RMSE	$\delta_{1}$	RMSE	$\delta_{1}$	RMSE
DA2	No Align	0.251	10.92	0.000	19.51	0.005	26.67	0.000	35.04	0.004	20.43
DA2	Disp-space	0.583	13.51	0.990	1.56	0.976	4.71	0.155	16.78	0.977	3.11
DA2	Depth-space	0.499	15.75	0.989	2.19	0.957	4.72	0.968	5.35	0.976	3.36
DA3	No Align	0.583	12.30	0.629	5.17	0.532	9.46	0.509	14.37	0.508	7.70
DA3	Disp-space	0.586	14.83	0.990	1.90	0.954	4.56	0.845	9.57	0.980	3.16
DA3	Depth-space	0.579	14.90	0.990	1.83	0.961	4.35	0.966	6.25	0.981	3.04
MoGe v1	No Align	0.000	16.70	0.000	27.51	0.000	43.19	0.000	63.87	0.000	29.89
MoGe v1	Disp-space	0.586	13.70	0.991	1.57	0.972	3.83	0.942	9.21	0.983	2.77
MoGe v1	Depth-space	0.578	14.46	0.992	1.76	0.969	3.85	0.967	5.14	0.983	2.88
MoGe v2	No Align	0.038	10.72	0.027	8.24	0.077	12.19	0.112	17.20	0.034	8.88
MoGe v2	Disp-space	0.585	13.77	0.990	1.59	0.965	4.09	0.910	10.13	0.981	2.85
MoGe v2	Depth-space	0.578	14.53	0.990	1.77	0.962	4.00	0.964	6.05	0.981	2.92

**Table 2:** Evaluation of 3D label quality using IoU and diagnostic KITTI-style AP.

Dataset	Methods ^†^	IoU(Threshold=0.5)			${\mathrm{AP}}_{3D}\;\left(\mathrm{IoU}=0.5\right)$		
		bbox(%)	bev(%)	3D(%)	Easy	Mod.	Hard
CamRadRoad	Deng et al. [30]	65.0	43.6	37.1	40.0	35.3	35.2
	Raw VFM [8] output	0.0	0.0	0.0	0.0	0.0	0.0
	VFM (Depth-aligned) [8] output	53.2	28.7	25.3	30.7	26.0	26.5
	VFM (Disparity-aligned) [8] output	51.5	25.3	26.2	25.8	23.2	20.0
	Ours (MA)	92.3	30.5	28.2	33.2	29.7	28.0
	Ours (MA + HF)	82.3	36.7	31.6	35.9	32.8	32.6
	Ours (MA + HF + CBA)	85.5	42.7	38.8	38.6	34.7	33.3
	Ours (MA + HF + CBA + SP)	84.5	49.1	43.0	47.5	43.5	43.0
V2X-Radar-I	Deng et al. [30]	68.5	48.8	35.9	42.8	38.4	37.9
	Raw VFM [8] output	0.0	0.0	0.0	0.0	0.0	0.0
	VFM (Depth-aligned) [8] output	58.6	35.4	24.8	33.1	29.2	28.7
	VFM (Disparity-aligned) [8] output	56.9	37.1	25.3	34.0	29.7	29.4
	Ours (MA)	90.8	35.9	25.4	34.5	30.8	29.9
	Ours (MA + HF)	82.1	43.5	31.0	39.4	36.3	35.1
	Ours (MA + HF + CBA)	83.4	50.6	37.6	43.2	38.7	38.4
	Ours (MA + HF + CBA + SP)	81.7	55.2	40.9	46.4	42.2	40.6

^†^ HF: heading fusion; MA: mask alignment with scale estimation; CBA: canonical bundle adjustment; SP: shape propagation. AP is reported only as a diagnostic label-quality metric because the auto-labeler does not output native confidence scores.

**Table 3:** Detector performance trained with pseudo-labels on the in-house roadside dataset and V2X-Radar-I. SE stands for semantic enhancement. All results follow the same train/validation split policy, IoU threshold, and KITTI-style R40 AP protocol.

Dataset	Detector	Modality	Labels	${\mathrm{AP}}_{\mathrm{BEV}}{\;\left(\mathrm{IoU}=0.5\right)|}_{R40}$			${\mathrm{AP}}_{3D}{\;\left(\mathrm{IoU}=0.5\right)|}_{R40}$		
				Easy	Mod.	Hard	Easy	Mod.	Hard
CamRadRoad	PVRCNN++ [4]	Point cloud	GT	51.3	33.1	33.1	48.4	28.9	28.9
			Ours w/o SE	48.4	28.9	28.9	46.3	26.4	26.4
			Ours w. SE	54.0	35.7	35.7	49.8	29.8	29.8
	CRN [21] Backbone	Radar	GT	56.3	49.8	49.7	53.3	44.5	44.5
			Ours w/o SE	52.9	50.0	49.9	50.0	43.4	43.3
			Ours w. SE	59.2	56.6	56.3	55.5	53.2	53.2
	Modified RCBEVDet [22,36]	Camera + Radar	GT	93.8	88.2	88.2	91.8	86.5	86.5
			Ours w/o SE	91.9	85.3	85.2	89.9	83.3	83.2
			Ours w. SE	96.2	90.1	90.4	92.1	87.1	87.2
V2X-Radar-I	PVRCNN++ [4]	Point cloud	GT	58.6	42.4	41.8	52.1	35.6	35.0
			Ours w/o SE	55.2	39.0	38.4	49.0	32.4	31.9
			Ours w. SE	61.8	45.7	44.9	53.6	37.0	36.8
	CRN [21] Backbone	Radar	GT	63.5	57.8	57.0	58.4	50.2	49.5
			Ours w/o SE	61.2	55.4	54.7	56.0	48.0	47.2
			Ours w. SE	66.4	61.1	61.3	60.7	54.6	53.5
	Modified RCBEVDet [22,36]	Camera + Radar	GT	95.0	90.5	90.0	92.0	86.8	86.2
			Ours w/o SE	93.2	88.1	87.5	90.0	84.2	83.6
			Ours w. SE	96.6	92.0	92.3	93.0	88.2	87.4

**Table 4:** Compact sun–night comparison on V2X-Radar-I. Label-quality values are estimated from the observed label-quality distribution; MoGe and mask-quality values are measured directly. VFM [8] output uses depth-space alignment.

Split	Label Quality		Aligned VFM [8] Output		2D Mask Quality	
	3D IoU (%)	${\mathrm{AP}}_{3D}$ Hard	$\delta_{1}$	RMSE (m)	Recall@0.5	Box IoU
Sun	41.0	37.6	0.912	5.96	0.8333	0.6796
Night	39.7	37.0	0.945	5.95	0.8750	0.6682
Δ N–S	−1.3	−0.6	+0.033	−0.01	+0.0417	−0.0114

## Data Availability

The data is unavailable due to privacy issues.
